# Developmentally regulated signaling pathways in glioma invasion

**DOI:** 10.1007/s00018-017-2608-8

**Published:** 2017-08-18

**Authors:** Shwetal Mehta, Costanza Lo Cascio

**Affiliations:** Division of Neurobiology, Barrow Brain Tumor Research Center, Barrow Neurological Institute, Phoenix, AZ 85013 USA

**Keywords:** Glioblastoma, Invasion, Migration, Cellular signaling, CNS development, Cancer stem cells, Cancer

## Abstract

Malignant gliomas are the most common, infiltrative, and lethal primary brain tumors affecting the adult population. The grim prognosis for this disease is due to a combination of the presence of highly invasive tumor cells that escape surgical resection and the presence of a population of therapy-resistant cancer stem cells found within these tumors. Several studies suggest that glioma cells have cleverly hijacked the normal developmental program of neural progenitor cells, including their transcriptional programs, to enhance gliomagenesis. In this review, we summarize the role of developmentally regulated signaling pathways that have been found to facilitate glioma growth and invasion. Furthermore, we discuss how the microenvironment and treatment-induced perturbations of these highly interconnected signaling networks can trigger a shift in cellular phenotype and tumor subtype.

## Introduction

Glioblastoma (GBM; World Health Organization grade IV glioma) is the most aggressive, infiltrative, and lethal brain tumor in adults [1]. These malignant tumors are currently incurable, due to their invasive nature and resistance to conventional therapies. Despite extensive molecular and genetic analyses into the biology of GBMs, patient outcome following standard of care therapies remains dismal, with a median survival of only 12–14 months [2]. One of the biggest challenges in the treatment of GBM is the presence of highly invasive tumor cells that disseminate into the normal brain parenchyma. These invasive cells evade surgical resection, resist conventional treatments that target proliferating cells, and are primarily responsible for tumor recurrence. Most recurrent GBMs display resistance to radiation and temozolomide, both of which are the first line of treatment for GBM patients following surgery [3]. Furthermore, it has been reported that GBMs frequently shift their biological features upon recurrence [4, 5]. For example, utilizing a murine model of a defined proneural GBM subtype, Halliday et al. demonstrated that radiation leads to a shift towards tumors with mesenchymal properties [6]. Similarly, anti-angiogenic therapies that have been approved for recurrent GBMs (avastin/bevacizumab) were also shown to generate aggressive and highly infiltrative tumors [7–10]. Since almost all GBM patients experience recurrence, effective clinical therapies are urgently needed to treat this malignancy.

**Fig. 1:**
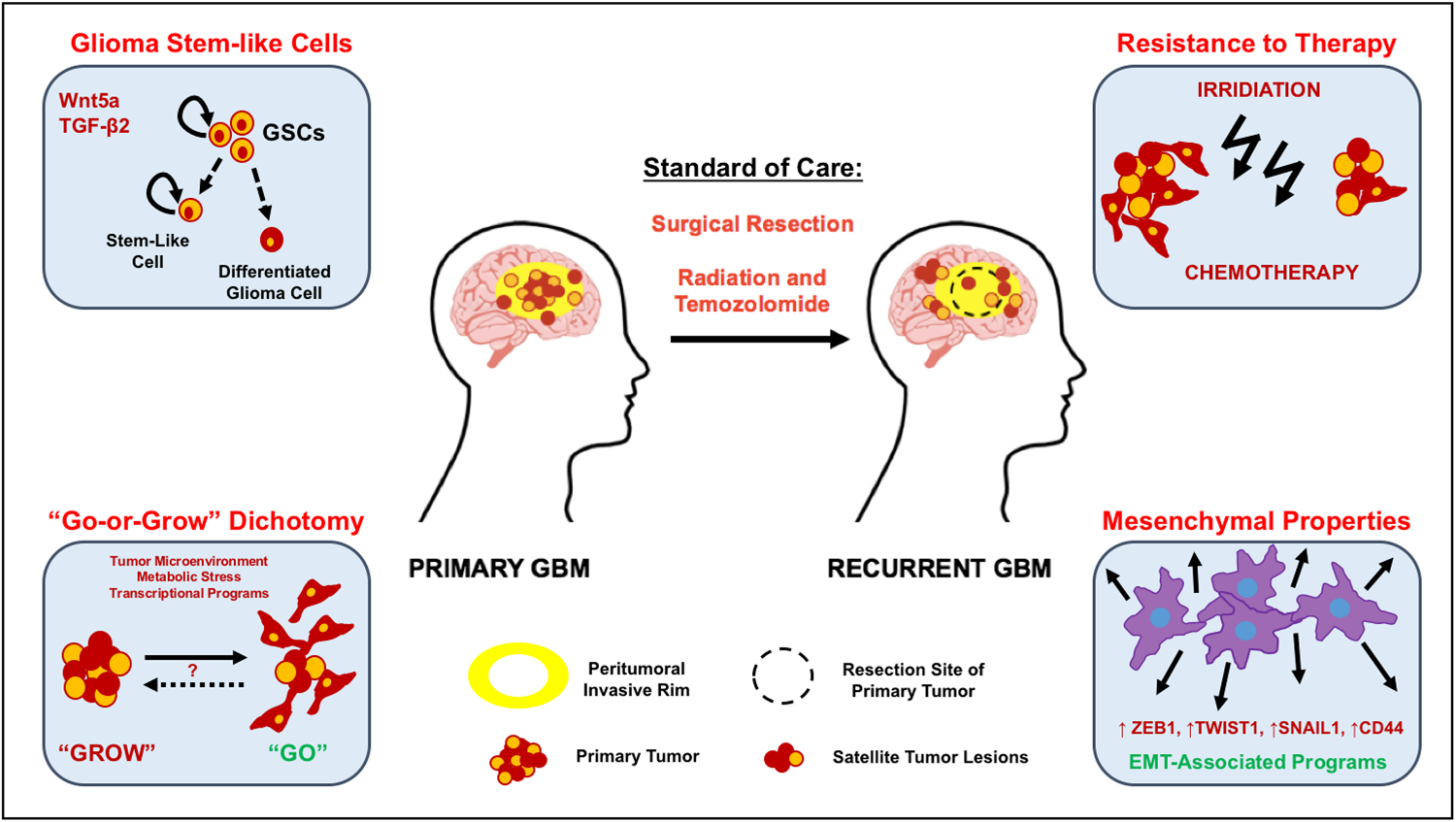
Molecular mechanisms that promote GBM invasion. GBMs are associated with high rates of mortality due to their intrinsic resistance to conventional therapies. Radiation treatment post-surgery, in conjunction with adjuvant chemotherapy (temozolomide), only leads to a modest increase in medial survival. Highly migratory glioma cells that are found in the peritumoral invasive rim evade surgical resection, resist conventional treatments that target proliferating cells, and inevitably lead to rapid tumor recurrence. Glioma stem-like cells (GSCs): the intrinsic resistance to genotoxic treatments manifested in GBM has been attributed to the presence of a rare subpopulation of cancer stem-like cells (*orange* cells) that harbor exclusive self-renewing and tumor-initiating potential. Several studies have shown that these GSCs display enhanced invasive behaviors in vitro and in vivo. Specifically, increased expression of Wnt5a and TGF-β2 have been found to enhance the invasion capacities of GSCs. The “Go-or-Grow” dichotomy: high-grade gliomas consist of a highly proliferative tumor core surrounded by a peritumoral zone of invasive cells, which are highly motile (*red* cells). Experimental evidence suggests that cell invasion and proliferation in glioma cells are mutually exclusive events, with proliferating cells being less migratory while rapidly migrating cells divide more slowly. Highly migratory cells escape surgical resection and invade the surrounding brain tissue, giving rise to satellite lesions that lead to tumor recurrence. It is likely that glioma cells revert between those two states at their convenience during tumorigenesis. Resistance to therapy: it has been proposed that cells with lower proliferation rates are less susceptible to conventional DNA-damaging agents. Hence, invasive, slowly proliferating cells that are left behind post-surgery are resistant to treatment and significantly contribute to tumor recurrence. Mesenchymal properties: numerous signal transduction pathways (WNT, RTK, and TGF-β) have been shown to modulate the expression of epithelial-to-mesenchymal transition (EMT)-related genes in glioma cells, inducing mesenchymal transformation and sustaining glioma cell dissemination into adjacent healthy brain tissue

Several genetic studies targeting specific cell populations in the brain suggest that the cell of origin for GBM is most likely a neural stem cell or stalled progenitor cell [11–13]. Moreover, these studies have also highlighted the parallels between normal neural development and gliomagenesis. During normal brain development, spatio-temporally regulated signal transduction pathways in neural stem cells ensure the generation of sufficient numbers of progenitor cells and their subsequent differentiation [14]. Large-scale genetic studies of more than 500 GBM tumors [15] and single cell RNA sequencing of glioma cells suggest that GBM cells utilize these same signaling networks to promote tumor growth and invasion [16–18]. In this review, we will examine our current understanding of the role of developmentally important signaling pathways whose normal function in neural stem/progenitor (NPC) cell proliferation and migration appear to be co-opted by malignant glioma cells to promote their rapid invasion into the surrounding brain parenchyma (Fig. 1).

## Cells on the right track: migration in the developing and adult CNS

Cell migration is an essential feature of newly formed neural and glial progenitors that ensures the formation of appropriate neuronal circuits and ensheathment of neurons. In the developing cortex, newly born neuronal and glial progenitors migrate from their site of origin to their distant functional locations throughout the central nervous system (CNS) [19, 20]. Radial glial cells that span all the way from the ventricular surface to the pial surface serve not only as a source of neuronal progenitors but also as a scaffold that aid the migration of newly formed neurons towards the cortical plate [21]. Oligodendrocyte progenitor cells (OPCs) are highly migratory cells that originate in the germinal areas of the neural tube and travel across the CNS to spread throughout both the gray and white matter [22]. In the adult brain, OPCs are the most abundant proliferative and migratory population. Upon brain injury, OPCs migrate to the site of injury and participate in the formation of ‘scar tissue’ [23]. The migratory properties of both glial and neuronal progenitors are regulated through concerted efforts of transcription factors, cell surface receptors, and extracellular cues [19, 20, 24, 25]. Glioma stem cells (GSCs) share many properties with NPCs, including the capacity to self-renew and ability to differentiate into various cells of the CNS. Elegant mouse modeling studies focused on cell of origin for GBMs suggest that upon perturbations of key signaling pathways both NSCs and OPCs are capable of giving rise to distinct subtypes of tumors [12, 13].

## Bad cells travel fast: the role of glioma stem cells in GBM invasion

There are numerous parallels between neurogenesis and the cellular processes that contribute to gliomagenesis. Hence, understanding the genetic basis of the development of these aggressive brain tumors is inextricably coupled with the study of the genes and signaling pathways that regulate cancer stem cell identity and biology [26]. Over the past decade, studies have shown that the intrinsic resistance to conventional treatments manifested in GBM can be attributed to a rare subpopulation of multipotent GSCs found in these tumors, which share many properties with normal NPCs [27–29]. As GSCs harbor exclusive self-renewing and tumor-initiating potential, they are believed to be the tumor-driving force in this fatal disease and play a significant role in tumor progression, maintenance, and recurrence after therapeutic intervention [30–32]. Importantly, several recent investigations have highlighted that this unique cell population may also be playing a previously underappreciated role in regulating the infiltrative nature of GBM [33–36].

Cancer stem cells have been reported to harbor increased invasive and metastatic potential across several malignancies [37–40]. In GBM, invading cells and GSCs share two common characteristics: they are highly resistant to radio- and chemotherapy and are often localized at the perivascular niche, in close contact with the endothelial cells [41, 42]. Critically, previous studies have shown that GSCs derived from human primary GBMs, GBM xenografts [30, 32], and brain tumor cell lines [43–45] display enhanced migratory and invasive behaviors in vitro and in vivo. It is likely that the invasive phenotype of GSCs is a natural extension of a NPC migratory program. Because of the ability of GSCs to survive conventional therapies—allowing them to initiate and sustain a new tumor [31]—and their potential to rapidly infiltrate and spread into healthy brain tissue, it is of special interest to understand how this rare cell population contributes to GBM invasion. This is particularly important considering that the cellular and molecular mechanisms through which GSCs are thought to drive GBM invasion are currently not well defined [46]. Furthermore, thorough investigation of these underlying mechanisms has the potential to provide novel insights that will be vital for the development of effective treatments for primary and recurrent GBM. Below, we review our present understanding of how two developmentally important signal transduction pathways are thought to orchestrate the invasive properties of GSCs.

### Wnt pathway

The Wingless/Int1 (Wnt) signaling pathway plays crucial roles at different stages of central nervous system (CNS) development, and is directly required for the regulation of self-renewal, proliferation and differentiation of NPCs in the developing brain [47, 48]. Aberrant activation of the Wnt pathway has been implicated in driving the formation and progression of various human cancers, most notably those occurring in the organs of the digestive tract, as well as GBM [49, 50]. Wnt signaling can be broadly classified into two separate pathways: the canonical (β-catenin dependent) or non-canonical (β-catenin independent) pathway. The canonical pathway has an established role in the maintenance and expansion of stem/progenitor pools, as well as lineage specification in both embryonic and adult tissues [51]. Conversely, the non-canonical Wnt pathway is an important regulator of cell movement and tissue polarity, and has been reported to control not only convergent extension movements during gastrulation but also neuronal and epithelial cell migration [52, 53].

Recently, Tsai et al. identified a role for the Wnt-Cxcr4 signaling pathway in regulating OPC–endothelial cell interactions, which was shown to be required for OPC migration along the vasculature and consequently their dispersal throughout the CNS [54]. With respect to gliomagenesis, there is abundant literature describing how the canonical Wnt/β-catenin pathway is essential to sustain the proliferation and self-renewal, and thereby the tumorigenic potential, of GSCs in high-grade gliomas [55–58]. Until recently, very little attention has been devoted towards understanding how key Wnt signaling components (particularly those involved in the migration-promoting non-canonical pathway) bestow GSCs with a highly invasive phenotype. Two recent studies have demonstrated for the first time that Wnt5a, a non-canonical Wnt ligand, appears to be a critical master regulator of the invasive capacity of human GSCs in vivo [33, 34].

Although aberrant expression or upregulation of Wnt5a has been associated with increased tumor cell invasion and metastasis across several different solid cancers [59–62], its role in the regulation of the invasive properties and progression of high-grade gliomas has not been fully delineated. While Wnt5a has indeed been shown to be overexpressed in GBM cells [63, 64] and has been implicated in modulating their migratory and proliferative abilities, these studies were conducted in immortalized glioma cell lines in vitro [64–66]. Recently, Binda et al. have shown that elevated levels of Wnt5a govern the infiltrative capacity of patient-derived GSCs using in vitro invasion assays, gene expression analyses and orthotopic xenograft mouse models of human glioma [33]. Critically, they found that Wnt5a expression levels in GSCs exhibiting a mesenchymal profile—which has been associated with reduced survival and increased invasive phenotype [67–69]—was 10- to 1000-fold higher compared to GSCs exhibiting a proneural or classical signature profile. Accordingly, a direct correlation was observed between Wnt5a expression levels and the inherent migratory/invasive potential in the human GSC lines employed in the study. Mesenchymal GSCs expressing high levels of Wnt5a activity displayed considerable invasive capability in vitro and in vivo [33].

These findings are consistent with a previous study demonstrating that non-canonical Wnt5a enhances migration of glioma cells by regulating the expression of matrix metalloproteinases (MMPs) involved in extracellular matrix (ECM) degradation [66]. Antagonizing Wnt5a activity in xenograft models through the use of Wnt5a-blocking antibody or a Wnt5a-derived hexapeptide hindered the aggressive and infiltrative behavior of these cells, resulting in reduced intracranial invasion and significantly increased survival relative to non-treated controls. Importantly, they show that overexpression of Wnt5a in classical GSCs triggered the acquisition of a highly migratory phenotype and an expression profile that matched the prototypical “invasive signature” of mesenchymal GBM. Hence, these results highlight that the switch to one subtype or another can be accomplished at the functional and molecular level—in this case by modulating Wnt5a expression in GSCs—and supports a role for Wnt5a as being a master regulator for determining the invasive potential of GSCs [33].

Utilizing a de novo model of GBM derived from immortalized human NPCs, Hu et al. proposed a novel mechanism by which Wnt5a could support GSC-mediated invasive growth [34]. Previous studies have observed that GSCs are enriched in perivascular and hypoxic niche, which have been shown to maintain the GSC pool and promote tumor progression and therapeutic resistance [41, 70]. Through transcriptomic and epigenomic analyses, Hu et al. demonstrated that human GSCs (expressing a constitutively active form of AKT and dominant-negative p53) utilize a Pax6/Dlx5 transcriptional program to regulate Wnt5a-mediated differentiation of GSCs into endothelial-like cells [34]. In turn, these GSC-derived endothelial-like cells produce Wnt5a and promote the recruitment and proliferation of host endothelial cells in a Wnt5a-dependent manner. This process facilitates the formation of peritumoral satellite lesions, thereby generating niches that support growth of invasive GBM cells away from the primary tumor in the surrounding brain parenchyma, ultimately leading to tumor recurrence [34, 71]. This is in line with previous studies wherein Wnt5a was shown to be required for the differentiation of embryonic stem cells into endothelial cells during vascular development, as well as endothelial cell proliferation, survival, and migration [72–74]. Considering that Dlx5 regulates Wnt5a expression during CNS development [75] and has been found to be expressed in GSCs [76], it appears that GSCs hijack this developmentally regulated Pax6/Dlx5-Wnt5A transcriptional axis to drive the differentiation of GSCs into endothelial-like cells, thus promoting the diffuse spread of infiltrative GBM cells. It is important to note, however, that GSCs have also been found to generate vascular pericytes that actively remodel the perivascular niches [77].

### TGF-β pathway

Another signal transduction pathway that has been found to contribute to the invasive nature of GSCs is the TGF-β pathway [78]. TGF-β signaling is a key player in a number of cellular processes regulating embryogenesis, cell proliferation, migration, and tissue homeostasis [79–81]. Although the TGF-β pathway is known for its tumor-suppressing function in epithelial tissues, it can also act as a promoter of tumorigenesis in various solid cancers—including GBM—due to its effect in enhancing cell migration, thereby promoting cellular invasion [82–88]. Elevated TGF-β signaling activity has been implicated in glioma pathobiology, with higher levels of TGF-β1 and TGF-β2 being found in GBM tumors compared to normal healthy brain tissue [85, 87, 89]. Moreover, high levels of *TGF*-*β2* expression have been associated with poor clinical outcome in GBM patients [90]. Mechanistically, TGF-β has been proposed to induce a mesenchymal phenotype in GBM cells through the activation of SMAD2 and ZEB1 (a TGF-β cytoplasmic signal transducer and a known transcriptional inducer of EMT, respectively, [91]) resulting in enhanced migration and invasion capacities of GSCs [92].

ZEB1 has previously been identified as being a key regulator of GSC invasion and stemness, upregulating not only epithelial-to-mesenchymal transition (EMT) genes but also critical GSC markers such as OLIG2 and SOX2 [93]. More recently, our group discovered that the unphosphorylated form of the neurodevelopmental transcription factor OLIG2 (essential for the gliomagenic properties of GSCs) induces invasion of patient-derived GSCs through the upregulation of the TGF-β2 signaling pathway, which in turn activates the expression of EMT-associated genes (*TWIST1*, *CD44*, *TGF*-*β2*, *CREB1*, and *ZEB1*) [36]. Importantly, evidence of the presence of ZEB1‐ and OLIG2- positive cells at the invasive front of GBM tumors strongly point to the existence of an invasive niche harboring GSCs [32, 36, 70, 93, 94].

## To proliferate or invade? Exploring the concept of “Go-or-Grow”

Uncontrolled proliferation and abnormal cell migration represent two of the main hallmarks of cancer [95]. High-grade gliomas consist of a highly proliferative tumor core surrounded by a peritumoral zone of invasive cells, which are highly motile and infiltrate the adjacent healthy brain parenchyma [96–98]. Expansion of this invasive front results in widespread diffusion of the tumor, posing a serious clinical challenge as this renders complete surgical removal of the tumor practically impossible [99]. Several key in vitro studies have demonstrated that glioma cells plated on a substrate exhibit different motility and proliferation rates [96, 100, 101]. Rapidly proliferating cells tend to be stationary, while actively migrating cells divide more slowly—suggesting that glioma cell invasion and proliferation are stochastically mutually exclusive events [96, 101–104]. Experimental evidence has also shown that glioma cells found within the bulky tumor core proliferate faster compared to cells at the peritumoral rim, which instead are slow growing and migrate at faster speeds [100, 105, 106]. Moreover, time-lapse imaging has revealed that glioma cells migrate in a saltatory fashion, wherein they pause close to vascular branch points to divide, and then resume migrating [107, 108]. This dichotomous relationship between proliferation and invasion is referred to as the “Go-or-Grow” hypothesis in the literature [100, 101, 107, 109].

Clearly, the intrinsic ability of glioma cells to sequentially shift their phenotype in response to treatment or metabolic stress has important implications for tumor progression and resistance to therapies [109]. For instance, it has been proposed that cancer cells with lower proliferation rates are typically less susceptible to conventional DNA-damaging agents [110]. It is possible that slow-proliferating, therapy-resistant, invasive glioma cells that escape surgical resection may later adopt (or revert to) a proliferative phenotype at satellite lesions, leading to rapid tumor recurrence. Hence, it is critical to fully elucidate the molecular mechanisms that modulate the switch between these two distinct cellular behaviors in GBM. Such studies will aid the development of innovative therapeutic strategies aimed at targeting both actively proliferating and migrating glioma cells.

**Fig. 2:**
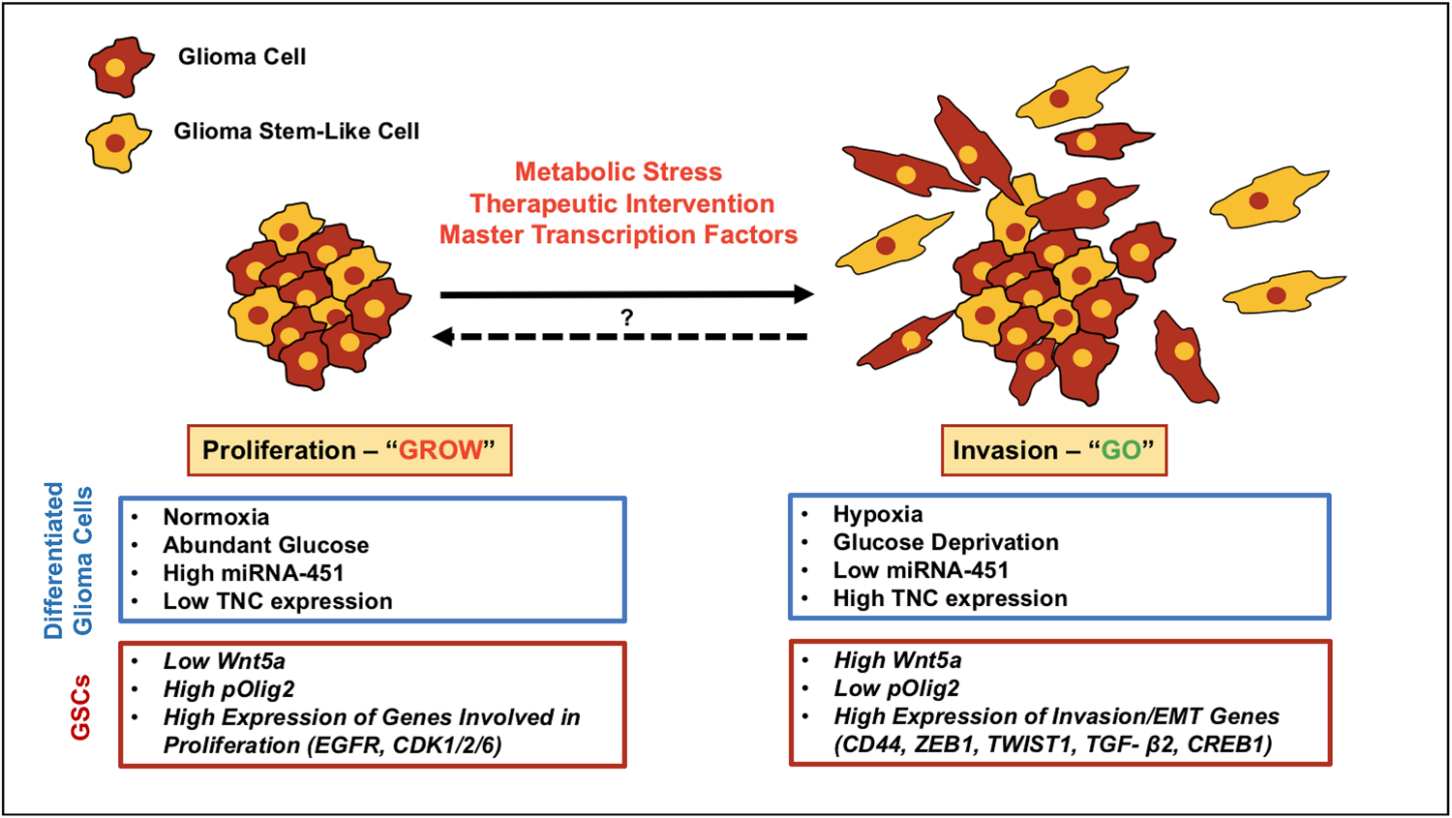
Exploring the “Go-or-Grow” dichotomy in malignant glioma. The dichotomous relationship between proliferation and invasion in GBM cells is referred to as the “Go-or-Grow” hypothesis. Genetic mutations might play a role in promoting glioma invasion; however, studies from several groups have shown that genes promoting invasion are upregulated, while proliferation genes are downregulated, in infiltrating/migrating tumor cells that are found at the peritumoral rim. Despite advances in our understanding of the cellular processes underlying glioma invasion, we know little about the molecular mechanisms that govern and regulate the switch between proliferation and invasion. Current evidence suggests that metabolic stress (hypoxia, glucose deprivation), the tumor microenvironment and the activation or suppression of key transcription factors may modulate the switch between these two distinct cellular behaviors in GBM. It is possible that therapy-resistant, invasive glioma cells (differentiated cells and GSCs) that escape surgical resection may later adopt (or revert to) a proliferative phenotype at satellite lesions, promoting rapid tumor recurrence. The *blue boxes* and *red boxes* highlight the driving forces that appear to regulate this molecular switch in differentiated glioma cells and GSCs, respectively

What is currently known about the potential mechanisms that modulate the emergence of an invasive tumor phenotype at the expense of a proliferative one? While it is possible that specific mutations may regulate this phenotypic switch, as shown in Fig. 2, the driving forces governing the antagonistic “Go-or-Grow” behavior in GBM have been primarily linked to metabolic stress, the tumor microenvironment and the temporal activation and/or suppression of key transcription factors [36, 104, 105, 111–114]. Additionally, several theoretical approaches—stochastic and probabilistic mathematical models—have been developed to study and recapitulate glioma growth dynamics in silico, allowing further analysis of the cellular and molecular processes underlying the “Go-or-Grow” dichotomy [106, 113, 115, 116].

### Metabolic stress

Cancer cells have the ability to resist adverse conditions such as hypoxia and metabolic stress, allowing them to influence numerous processes that are required to support tumor growth, such as migratory capacity, proliferation, survival, and angiogenesis [117]. A series of studies conducted by Godlewski et al. demonstrated that a single miRNA, miRNA-451 controls the balance between cell proliferation and migration in different glioma cell lines in response to glucose deprivation [111, 112]. In a rapidly growing tumor such as GBM, cancer cells are often under metabolic stress because the available glucose levels tend to fluctuate, meaning that these cells must adopt alternative strategies to ensure an adequate glucose supply to sustain tumor cell growth [118]. Under conditions where glucose is abundant, miRNA-451 is highly expressed in glioma cells and promotes proliferation, while low glucose levels lead to the downregulation of miRNA-451, resulting in enhanced glioma cell migration and allowing survival at the expense of proliferation [111]. miRNA-451 is a negative regulator of the LKB1/AMPK pathway, which is normally activated when cells undergo periods of low energy availability [119]. Reduced glucose conditions cause miRNA-451 levels to decline in glioma cells, and consequent activation of the LKB1–AMPK pathway promotes increased cell migration and invasion through the activation of cytoskeletal proteins, while reducing cell proliferation by inhibiting mTOR activity [111, 112].

Similar to miRNA-451, another study found that carboxypeptidase E (CPE), a secreted neuropeptide-processing enzyme, also possesses an anti-migratory and pro-proliferative capacity, modulating the growth behavior of glioma cells in response to hypoxia and glucose deprivation [99, 114]. Moreover, investigations aimed at understanding how GSCs adapt to bidirectional fluctuations in oxygen revealed that glucose metabolic pathways are indeed causatively involved in the dichotomous regulation of the “Go-or-Grow” cellular program [120, 121], supporting the long-standing view that GSCs harbor outstanding metabolic adaptability [122, 123].

### The tumor microenvironment

It is now well accepted that interactions between glioma cells and the surrounding normal brain microenvironment modulate the proliferative and invasive properties of glioma cells. A recent study by Xia et al. proposed that the extracellular matrix glycoprotein tenascin C (TNC), which mediates cell–cell and cell–ECM interactions, can regulate the “Go-or-Grow” phenotypic switch of GSCs in vivo [104]. TNC is predominantly expressed during embryogenesis [124] and within the NPC niche [125], but it is also highly upregulated in the microenvironment of various malignancies, including GBM [104, 126]. Using intracranial xenograft mouse models, the authors revealed that TNC knockdown stalled tumor invasion and increased tumor cell proliferation [104]. Although the molecular mechanisms mediating these effects are not entirely clear, the activity of the PI3K/AKT, MAPK, and FAK pathways (all of which promote cell proliferation and/or adhesion) increase upon TNC silencing, implicating that this ECM protein may be involved in the maintenance of the GBM invasive niche and promotion of tumor cell invasion [104, 127]. These studies highlight how microenvironmental factors and responses that allow glioma cells to thrive under metabolic stress can influence their decision to either migrate (“go”) or proliferate (“grow”) to better adapt to their local surroundings, and thereby sustain tumor progression.

In addition to the ECM-derived factors that promote invasion, glioma cells receive pro-migratory signals from infiltrating microglial cells—the resident macrophages of the brain—and peripheral macrophages [collectively known as the tumor-associated macrophages (TAMs)]. Glioma cells secrete several factors that serve as a chemoattractants for microglial cells, such as the hepatocyte growth factor (HGF), colony-stimulating factor 1 (CSF-1), monocyte chemoattractant protein-1 (MCP-1/CCL2), stromal cell-derived factor-1 (SDF-1), and glial-derived neurotrophic factor (GDNF) [128–131]. Several studies have demonstrated that these TAMs can promote glioma growth and invasion; however, it is important to note that most of these investigations were conducted using immortalized human glioma cells or syngeneic mouse models. Nevertheless, experimental evidence has revealed that GSCs harbor increased capability of attracting TAMs compared to differentiated glioma cells [132]. Furthermore, TAMs have been shown to increase the invasive potential of GSCs by stimulating the secretion of MMP-9 in a TGF-β-dependent manner [133]. For a more detailed review on the role of TAMs in the promotion of glioma cell invasion, please refer to Hambardzumyan et al. [134].

### Ion channels in glioma invasion

In conjunction to inducing extensive remodeling of the ECM, migrating glioma cells have also been shown to undergo dramatic changes in shape and volume to further facilitate their movement through the very narrow and tortuous extracellular spaces of the brain. To be able to fit and navigate through those limited extracellular spaces, invading glioma cells dramatically shrink their cell volume and acquire an elongated shape [135] [136]. Invasive glioma cells can reduce their cell volume by shedding their cytoplasmic content [137]. This process requires the concerted efflux of K^+^ and Cl^−^ ions, which consequently forces water to passively leave the cell by flowing down its osmotic gradient [138, 139]. This mechanism, which has been previously referred to as the hydrodynamic model of cell invasion [136, 138], postulates that glioma cells repurpose K^+^ and Cl^−^ ion channels to regulate their cytoplasmic water content and thereby enable adjustments in cell shape and volume that are necessary for cell invasion [138].

Among various ion channels, glioma cells express the voltage-gated Cl channel protein 3 (ClC3) and the Ca^2+^-activated K^+^ channels KCa1.1 and KCa 3.1 [139]. Stimulation of G protein-coupled receptors (GPCRs), notably the bradykinin 2 receptor (B2R), and the RTK EGFR can induce inositol-1,4,5-trisphosphate receptor 3 (IP3R3)-dependent increases in intracellular Ca^2+^ concentration in glioma cells [138, 140]. Elevated Ca^2+^ levels, in turn, lead to KCa3.1 and KCa1.1 channel opening and activate Ca^2+^/calmodulin-dependent protein kinase II (CaMKII), which phosphorylates and activates ClC3 [138, 139]. This cascade results in a simultaneous efflux of K^+^ and Cl^−^ ions and obligates water to flow out of migrating glioma cells, which are then able to decrease their cytoplasmic volume and squeeze through narrow spatial barriers in the cerebral parenchyma.

During development, ion channels play an important role in the regulation of cell division, differentiation, and migration through changes in membrane potential [141]. In line with the “Go-or-Grow” hypothesis, hyperpolarization of the membrane induces cell differentiation, while depolarization activates cell proliferation. Not surprisingly, the expression and activity of K_v_ channels are regulated in a cell cycle-dependent manner both in normal and cancer cells [142, 143]. Moreover, inhibition of K_v_ channel isoforms in neural stem cells has been shown to reduce proliferation of neurospheres in vitro [144, 145].

Several studies have also revealed important roles for glutamate in glioma biology, and evidence suggests that glutamate may serve as a key autocrine signal that enhances glioma cell invasion [138, 146–148]. Glutamate is produced from glutamine by glioma cells and is released through the system x_c_^−^ cysteine–glutamate antiporter, which is highly expressed in human gliomas [149, 150]. Apart from inducing neuronal hyperexcitability and neurotoxicity, high extracellular levels of glutamate have been shown to promote tumor invasion by binding to and activating Ca^2+^-permeable AMPA receptors (AMPAR) on glioma cells [146, 147]. Glioma cells express a variant of the AMPAR that lacks the GluR2 subunit, which when present would normally render AMPARs impermeable to Ca^2+^ ions [146, 147, 151]. Glutamate-induced activation of Ca^2+^-permeable AMPA receptors on glioma cells generates oscillations in intracellular Ca^2+^ concentration, a process which has previously been shown to be required to drive the migration of invading glioma cells [147]. Moreover, inhibition of either Ca^2+^-permeable AMPA receptors or x_c_^−^-mediated glutamate release in glioma cells resulted in disruption of glioma cell migration and generation of less-invasive tumors in vivo [146, 147]. All these observations imply a dual role for tumor-derived glutamate in glioma invasion: it not only acts in an autocrine fashion to stimulate tumor cell motility but also its toxic extracellular concentrations may help promote tumor expansion by killing normal cells in the surrounding brain parenchyma, generating corridors that allow glioma cells to leave the primary tumor and spread.

### Key transcription factors

There is emerging evidence that the dichotomy between cell proliferation and cell migration in GBM cells may also be dictated by the temporal activation and/or suppression of specific transcription factors (TFs). Studies from several groups, including ours, suggest that genes promoting invasion are upregulated, while proliferation genes are downregulated, in infiltrating, migrating tumor cells [36, 105, 152, 153]. Transcriptional profiling of laser capture-microdissected GBM cells collected from matched patient tumor core and invading rim regions previously indicated that these tumor cell subpopulations harbor distinct gene expression signatures [154]. For example, Dhruv et al. reported that increased NF-κB activity was observed in radially dispersed, invading glioma cells, while high c-Myc activation was detected in migration-restricted, proliferative cells within the tumor core [105]. This difference in gene expression points at a possible key role for TFs in the modulation of the phenotypic behavior of glioma cells, especially considering that numerous TFs (e.g., ZEB1, STAT3, C/EBPb, and TAZ) have been shown to play a critical role in the induction of invasive mesenchymal phenotypes in GBM cells [68, 93, 155].

Our group recently demonstrated that the phosphorylation status of a CNS-specific TF, OLIG2, acts as a molecular switch that regulates the transition from a proliferative to an invasive phenotype in GSCs [36]. OLIG2 is a basic helix–loop–helix (bHLH) protein expressed in the multipotent NPCs in the developing brain, and is required for the generation of oligodendrocytes and certain subtypes of motor neurons [156]. OLIG2 has been shown to be universally expressed in almost all diffuse gliomas [157, 158], and its expression is required for gliomagenesis in a genetically relevant murine model and in orthotopic patient-derived xenograft models [159, 160]. While the phosphorylated form of OLIG2 is essential for glioma cell proliferation [161], we discovered that unphosphorylated OLIG2 promotes glioma invasion through upregulation of the TGF-β2 signaling pathway [36]. In murine and patient-derived GSCs, the levels of phosphorylated OLIG2 are inversely correlated with invasive capacity and directly correlated with proliferation. Interestingly, we found that overexpression of either a phospho-mimetic or phospho-null mutant of OLIG2 in GSCs can induce a switch to a proliferative or migratory phenotype and vice versa in vitro.

OLIG2 expression has been previously linked to migration/invasion, both in normal and in malignant cells [93, 153, 162], and OLIG2-expressing cells have been found both at the GBM tumor core and peritumoral rim [36, 163]. Furthermore, OLIG2 is expressed in OPCs, the major proliferating cell population in the adult brain [159, 164], and has been implicated in regulating OPC migration [162]. Hence, our data strongly suggest that GSCs most likely exploit OLIG2’s normal role in OPC migration to invade and populate the normal brain parenchyma during tumorigenesis. Thereby, our experimental findings provide significant mechanistic insight into how the post-translational modifications of a single TF can reversibly enhance either tumor cell growth or invasion. What signals (intrinsic as well as extrinsic) trigger the modulation of OLIG2 phosphorylation remains an open question that warrants further investigation.

## Breaking free: pathways regulating the epithelial-to-mesenchymal transition in GBM

Epithelial-to-mesenchymal transition (EMT) is a multistep biological phenomenon by which polarized epithelial cells undergo numerous biochemical alterations that ultimately result in the loss of epithelial organization and the acquisition of a mesenchymal phenotype, which endows cells with increased motility and reduced intercellular adhesion [165]. EMT is indispensable during embryonic development, in processes such as gastrulation and neural crest formation, and is also critical during wound healing and tissue remodeling [166, 167]. It is currently widely established that carcinoma cells co-opt this mechanism to drive cancer cell invasion and metastasis to distant organs [168, 169].

Two critical hallmarks of EMT include detachment of epithelial cells from the basement membrane [166], and a switch from E-cadherin to N-cadherin expression [170]; however, the adult CNS lacks basement membranes outside of the vasculature [171] and the expression of E-cadherin in the brain is very rare [172]. Despite these important differences, there is ample experimental evidence suggesting that the same crucial intracellular effector molecules and master TFs that mediate EMT in epithelial cancers can also induce mesenchymal features in GBM [173], although the relevance of EMT-like processes in malignant brain tumors remains controversial [174]. GBMs belonging to the mesenchymal subtype are characterized by very poor clinical prognosis compared to other subtypes, significantly shortened time to recurrence following initial treatment, elevated invasive potential and increased aggressiveness [68, 69, 77, 175]. While the study of the role of EMT-like mechanisms in GBM has continued to receive little attention in the past few years, a number of investigations have elucidated how interconnected signaling pathways and their downstream transcriptional regulators putatively regulate and induce the acquisition of mesenchymal phenotypes in GBM, highlighting similarities with other solid cancers [93, 155, 176–181].

**Fig. 3:**
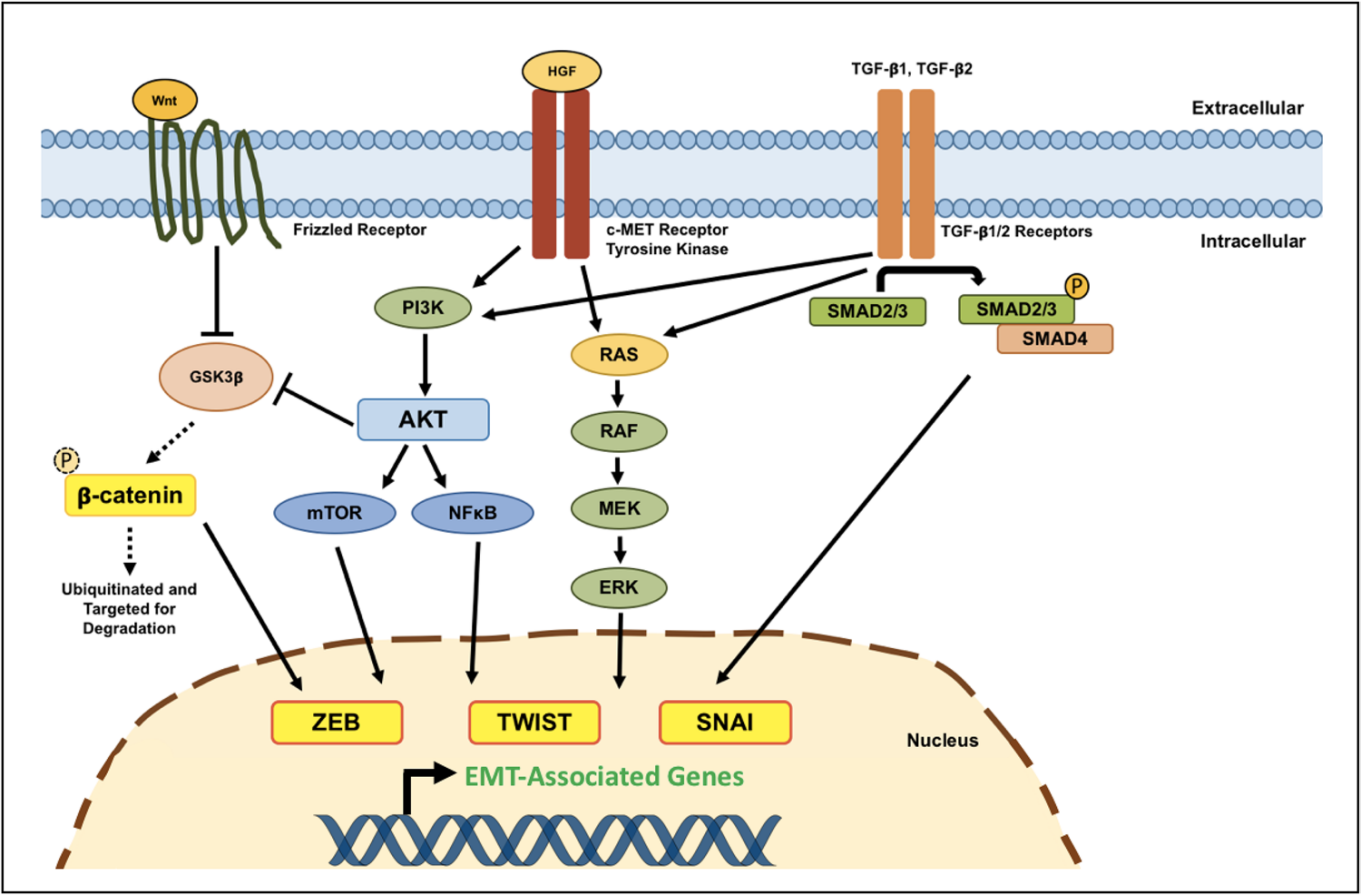
Molecular mechanisms regulating EMT-like programs in GSCs. Several interconnected signaling pathways have been shown to activate EMT-like processes in GBM, promoting the acquisition of a mesenchymal phenotype in GSCs. Signaling pathways whose deregulation/aberrant activity has been found to promote the expression of EMT-associated genes in GBM primarily include the Wnt/β-catenin, hepatocyte growth factor (HGF)/c-MET, and TGF-β pathways. Canonical Wnt/β-catenin pathway: in the absence of canonical Wnt signals, GSK3β readily phosphorylates cytoplasmic β-catenin, targeting it for subsequent degradation through the ubiquitin–proteasome pathway. However, when Wnt ligands bind to a Frizzled receptor, GSK3β activity is inhibited (although this inhibition can also be mediated through the PI3K/AKT pathway). Active Wnt signaling thereby allows β-catenin to accumulate in the cytoplasm, which can subsequently translocate into the nucleus and bind to LEF/TCF transcription factors, in doing so activating the expression of target EMT genes. HGF/c-MET pathway: the c-MET receptor tyrosine kinase is often aberrantly overexpressed in GBM cells, including glioma stem-like cells, and has been implicated in enhancing tumor invasiveness by modulating the expression of genes involved in EMT. Upon binding its cognate ligand, HGF, c-MET can activate the RAS/MAPK and PI3K/AKT signaling cascades, both of which have been shown to lead to increased expression of transcription factors involved in glioma cell motility and invasion. TGF-β pathway: binding of TGF-β1 or TGF-β2 to either type I/II TGF-β receptors induces phosphorylation, and thereby activation, of receptor-regulated SMAD proteins (SMAD2/3). Activated SMAD2/3 is then able to interact with the common-mediator SMAD4, forming a trimeric SMAD complex that is free to translocate into the nucleus and cooperate with transcription factors to induce the expression of EMT-related genes. Moreover, TGF-β signaling can also induce EMT through SMAD-independent pathways through activation of the MAPK and PI3K/AKT signaling cascades, but these mechanisms have not been studied extensively in the context of GBM invasion

EMT is principally orchestrated by three families of TFs: SNAI, ZEB, and TWIST [182]. In multiple human epithelial cancers, the upregulation of these TFs is associated with enhanced tumor invasiveness, worse clinical prognosis and collectively they are considered to be one of the main driving forces behind the metastatic cascade [183–186]. Increased activity of these three TFs has also been extensively reported in GBM—relative to levels found in normal brain tissue—and has indeed been found to promote glioma cell migration and invasion [93, 176, 178, 180, 187–189]. Below, we discuss how various cellular signaling pathways modulate the expression of these master EMT-related genes to sustain GBM cell dissemination, as well as how therapeutic interventions are capable of inducing mesenchymal transformation in GBM (Fig. 3).

### Wnt/β-catenin pathway

The canonical Wnt/β-catenin pathway has an established role in the induction of EMT, has been shown to enhance the motility of breast, colon, and pancreatic cancer cells [190–192]. With respect to high-grade gliomas, a study by Kahlert et al. found that the Wnt/β-catenin pathway was predominantly activated (as assessed by β-catenin nuclear staining) within cells located at the invasive peritumoral front of 30 GBM patient specimens belonging to the mesenchymal subclass [178]. Mechanistically, binding of canonical Wnt ligands to Frizzled (FZD) receptors results in the activation of the canonical Wnt pathway, leading to the inhibition of GSK-3β which consequently fails to phosphorylate β-catenin and hence targets it for ubiquitination [165]. Therefore, active canonical Wnt signaling allows cytoplasmic accumulation of β-catenin, which is then free to translocate into the nucleus and regulate the expression of its target genes. Accordingly, active Wnt/β-catenin signaling in primary GBM-derived cultures induced the expression of key EMT activators, including ZEB1, TWIST1, SLUG1, and enhanced the migratory capacity of the cells in vitro, whereas silencing β-catenin through RNA-interference decreased the expression of these EMT-related TFs and abrogated glioma cell invasion [178].

Another study revealed that a highly invasive glioma cell line, U87R4, harbored increased expression of the FZD4 receptor, which was found to promote the acquisition of a mesenchymal phenotype (*CD44* and *SNAI1* expression) [55]. More recently, Wnt/β-catenin signaling was implicated in upregulating the expression of Fos-related antigen-1 (Fra1), a gatekeeper of the EMT program in various cancers [193, 194], which was found to promote resistance against the chemotherapeutic agent cisplatin in vitro and in vivo [181]. These findings warrant further investigation considering that active Wnt/β-catenin signaling has previously been statistically associated with decreased GBM patient survival [195].

### TGF-β pathway

The TGF-β pathway is known to play a predominant role in the activation of EMT programs across various malignancies [165]. Binding of TGF-β family proteins to tetrameric complexes of type I or II TGF-β-receptors (TβRI and TβRII) can induce EMT through either SMAD-dependent or SMAD-independent signaling events [196]. Although there is ample evidence demonstrating that the TGF-β pathway is critical for the promotion of invasive mesenchymal properties of glioma cells [85–87, 197], studies have only recently started to dissect the molecular mechanisms through which TGF-β signaling drives the expression of EMT-related genes in GBM [174].

TGF-β signaling has previously been implicated to be critical for the maintenance of the mesenchymal stem-like population in GBM [197]. We have shown that OLIG2-dependent upregulation of TGF-β2 signaling in human GSCs triggers TβRI/II-mediated phosphorylation of SMAD2, resulting in the induction of the expression of various genes known to be important for EMT (*TWIST1, CD44, TGF*-*β2, CREB1*, and *ZEB1*) [36]. Receptor-regulated SMAD proteins (SMAD2/3) are activated through phosphorylation, and can subsequently associate with SMAD4 to form a trimeric co-regulatory complex that translocates into the nucleus to activate its downstream genetic targets [198]. In agreement with our results, Nevo et al. found that silencing *OLIG2* resulted in decreased expression of the EMT gene *TWIST1* [153]. Furthermore, we demonstrated that ZEB1 is a direct genetic target of OLIG2, hinting at a cross-regulatory loop wherein unphosphorylated OLIG2 leads to an increase in ZEB1, which in turn upregulates OLIG2 expression [36].

Our findings are also in line with a previous study that revealed that TGF-β signaling enhances the migratory capacity of immortalized GBM cells, promoting a mesenchymal shift in vitro through the activation of SMAD2 and ZEB1 [177]. Intriguingly, while exposure to TGF-β and subsequent activation of SMAD2 led to a significant change in the cellular morphology (characterized by a more stretched and elongated appearance) and an enhanced scattered growth pattern of treated U87 and U251 cells, the expression of critical EMT TFs other than ZEB1—such as SNAIL1, SNAIL2, and TWIST—were not significantly upregulated [177]. Specifically, accumulation of nuclear ZEB1 was correlated with enhanced expression of two mesenchymal markers, collagen 5A1 (COL5A1) and fibronectin, as well as elevated invasive potential of glioma cells both in vitro and in vivo [177, 199].

It is important to highlight that TGF-β can also contribute to the induction of EMT through signal transduction cascades that do not involve the SMAD proteins, such as the RHO-like GTPases, PI3K/AKT/mTOR, and MAPK pathways [165, 200, 201]. Surprisingly, compared to other cancers, very little work has been devoted to studying the role of TGF-β-induced EMT through non-SMAD pathways in the context of malignant gliomas [196]. While there is sparse evidence in the literature pointing at a perhaps underappreciated involvement of TGF-β-activated PI3K/AKT and MAPK signaling in the regulation of EMT-like programs in GBM [174, 202–205], further experimental work is required to precisely define how these pathways promote the emergence of mesenchymal phenotypes in glioma cells.

### Hepatocyte growth factor/c-MET signaling

c-MET is a tyrosine kinase receptor that binds a single pleiotropic growth factor, HGF [206], and is a proto-oncogene that activates a wide range of intracellular signaling pathways involved in the promotion of cell proliferation, angiogenesis, migration, and survival across different organs [207]. HGF/c-MET signaling is critical for morphogenetic processes that occur during embryogenesis, as well as wound healing and adult tissue regeneration [207–209]. Upon HGF binding, c-MET activates several downstream signaling cascades to induce EMT, primarily the PI3K/AKT, RAS/MAPK, and Wnt/β-catenin pathways [210, 211]. Aberrant c-MET activity, arising due to either gene mutation or amplification, has frequently been implicated in the development and progression of multiple human cancers, including high-grade gliomas [212].

With respect to GBM, c-MET has been shown to be particularly overexpressed within GSC populations [213] and in patient-derived GSCs exhibiting a mesenchymal subtype gene expression profile [214]. Accordingly, elevated c-MET signaling was previously found to enhance GSC migration by activating EMT TFs [214, 215] and endow GSCs with therapeutic resistance by promoting cell survival [216]. Moreover, oncogenic c-MET signaling is associated with poor survival and increased tumor invasiveness in GBM patients [217–219]. Considering that it has been postulated that HGF/c-MET concomitantly regulate both EMT-associated programs and stemness features in GSCs [217], this pathway not only appears to significantly contribute to invasive glioma growth but also sustains the glioblastoma stem cell phenotype that is thought to drive tumor recurrence [206].

### Therapy-induced mesenchymal transformation in GBM

Several recent studies have revealed that treatment of primary GBMs with radiation therapy or anti-angiogenic agents (bevacizumab) promote the acquisition of an aggressive treatment-resistant, or mesenchymal, phenotype in recurrent tumors [6, 7, 10, 179, 202]. There is emerging evidence that malignant cells that survive these treatment modalities strongly upregulate pathways that promote the induction EMT, thereby resulting in increased tumor cell invasion [173].

With respect to radiation-induced EMT, it has been repeatedly shown that treating malignant glioma cells with sub-lethal doses of radiation in vitro significantly enhances tumor cell motility through upregulation of TGF-β, HGF/c-MET, and vascular endothelial growth factor (VEGF) signaling [220–222]. This is consistent with other studies that have found that glioma cells that have acquired radioresistant properties post-treatment exhibit a gene expression profile enriched for genes involved in EMT-related processes, which consequently contribute to spearheading highly invasive growth patterns upon tumor recurrence [202, 223, 224]. Furthermore, an in vivo study by Halliday et al. demonstrated that proneural tumor cells in a PDGF-driven mouse model of glioma clearly and rapidly shifted their expression pattern towards a mesenchymal one in response to radiation treatment [6]. As radiation is a universal component in the treatment of GBMs, this subtype shift poses an important clinical challenge, especially considering that it has also been shown that proneural glioma cells that have shifted to a mesenchymal subtype display increased radioresistant phenotypes [155].

There is also evidence that GBM tumors quickly acquire adaptive resistance to anti-angiogenic therapies such as Bevacizumab, a humanized monoclonal antibody against VEGF, and transition to a more invasive phenotype [10, 179, 225]. Considering that GBMs are highly vascularized tumors and express elevated levels of VEGF [226, 227], there is potential value in developing treatments that target angiogenesis [7]. However, while Bevacizumab was shown to prolong progression-free survival in patients with newly diagnosed GBMs, treatment failed to prolong overall survival [228, 229]. The initial beneficial effects of bevacizumab are only transient given that GBM tumors—in a similar manner to radiation therapy—inevitably recur during treatment by employing alternative pathways that sustain tumor growth when VEGF signaling is inhibited [173]. Other studies have also shown that tumors develop progressive hypoxia following Bevacizumab treatment, which consequently directly or indirectly promotes the emergence of a mesenchymal phenotype [10, 230, 231].

Using an orthotopic xenograft model of human glioma, Lu et al. demonstrated that VEGF suppresses HGF-mediated c-MET activation (which, as explained earlier, promotes tumor cell migration) [179]. When VEGF activity is genetically or pharmacologically inhibited, the resulting tumors were largely non-angiogenic, highly diffuse and the infiltrating tumor cells harbored high levels of phosphorylated (active) c-MET [179]. This study revealed that VEGF blockade restores and promotes c-MET activity in invading glioma cells, leading to the upregulation EMT genes (Snail, N-Cadherin) and enhanced mesenchymal features in a hypoxia-independent manner [179]. Such results indicate that increased c-MET activity in response to anti-VEGF therapy potentially leads to the emergence of a pro-invasive phenotype in GBM patients treated with Bevacizumab.

One of the challenging questions in the field is whether the shift in cellular phenotype and tumor subtype is due to cell extrinsic changes in the microenvironment or due to clonal selection of a mutant, therapy-resistant glioma cell. Recent genomic profiling of temozolomide-treated and untreated low grade gliomas [232] and single cell RNA-seq analysis from GBM tumor tissues [18] suggest that both these scenarios possibly regulate shifts in cellular phenotype.

## Paving the way for invasion: how glioma cells remodel the ECM

Unlike other malignancies, GBM cells very rarely metastasize to other organs in the body [233]. In GBM, glioma cells migrate through two types of extracellular spaces: the brain parenchyma, which constitutes the white matter tracts as well as the interstitial spaces between neurons and glial cells, and the perivascular spaces that surround the walls of blood vessels [46]. To ensure tumor dissemination into the surrounding healthy brain tissue, glioma cells located at the invasive front must undergo several complex biochemical and morphological changes. Such processes entail detachment from the tumor mass, acquisition of a highly migratory phenotype, and interactions with, as well as degradation of, multiple protein components of the brain ECM (a complex mixture of glycosaminoglycans, laminin, fibronectin, tenascin, nidogen, fibrillar collagens, and elastin) [174, 234–236]. Although the ECM is clearly a physical barrier that glioma cells must pass through to establish non-delineated paths for invasion, it also provides the appropriate ligands (e.g., integrins, tenascin C) that glioma cells can transiently utilize as anchors to pull themselves forward [237]. Hence, apart from establishing physical interactions with the ECM to their advantage, invading glioma cells must also be capable of chemically remodeling the overall structure and composition of the brain ECM to allow widespread tumor growth [237, 238].

At the leading edge of most solid cancers, including GBM, complex proteolytic events mediated by proteinases and/or proteinase activators expressed in invading tumor cells play a significant role in directing cell migration through the ECM [46, 236]. Indeed, numerous studies have reported that glioma cells overexpress many ECM-degrading proteinases, including various matrix metalloproteinases (MMPs, MMP-2, MMP-9, MMP-13), metalloendopeptidases (ADAMs), cysteine proteinases (cathepsin B, L, S), aspartic proteinases (cathepsin D), and serine proteinases (urokinase-type plasminogen activator) [237, 239–243]. Since extensive ECM remodeling is a necessary step for tumor cell invasion, it is not surprising that the same signaling pathways we have mentioned throughout this review (WNT, PI3K, MAPK, HGF/c-MET, TGF-β) have also been found to significantly upregulate the expression of a myriad of proteolytic enzymes in malignant glioma [46, 66, 133, 244–247]. For a more elaborate discussion pertaining to how glioma cells actively employ all these protease families to facilitate rapid invasion into the brain parenchyma, please refer to an excellent recent review by Sayegh et al. [248].

Hyaluronic acid (HA), a glycosaminoglycan that regulates cell adhesion and migration, has also been implicated in providing microenvironmental cues that promote the infiltration of invasive glioma cells through the surrounding ECM [249]. Importantly, HA is found to be more abundant in the peritumoral regions compared to distant normal brain tissue [250]. A recent study by Lim et al. suggests that tumor-associated mesenchymal stem-like cells—stromal cells that interact with glioma cells—may contribute to the high abundance of HA in the tumor microenvironment through the induction of HA synthase-2 [251]. Apart from providing mechanical support, HA interacts with multiple cognate receptors—including CD44, receptor for hyaluronate-mediated motility (RHAMM), and intercellular adhesion molecule-1 (ICAM-1)—whose elevated expression correlate with worse patient prognosis in GBM [252]. Moreover, interactions between HA and CD44 and RHAMM in GSCs have been shown to result in the activation of intracellular signaling cascades that enhance both self-renewal and tumor cell invasion [253–255].

## Additional pathways involved in glioma invasion

Several recent investigations have provided important insights with respect to additional cellular pathways (Integrin/FAK, TWEAK-Fn14, Rho/ROCK) that appear to specifically promote and sustain the invasive properties of GSCs. However, while these pathways have been previously extensively studied in the context of glioma invasion, it is worth highlighting that these experiments were conducted using serum-grown immortalized cells (e.g., U87 or T98G). Since our goal in this review is to provide a comprehensive review of the mechanism driving invasion in GSCs, we have focused our discussion on recent studies that have employed human GSCs.

## The road ahead: closing remarks and future prospects

How do these studies inform the ongoing clinical care of GBM patients? A number of targeted small-molecule inhibitors targeting active kinases (e.g., EGFR, PDGFRa, PI3K, etc.) or MMPs [256] have been evaluated as single agents or in combination with standard of care for recurrent and newly diagnosed GBM. However, none of these trials have improved progression-free survival or overall survival in GBM patients so far. With the recent advances in immunotherapy, various immunomodulatory approaches are being evaluated in glioma clinical trials, including antibodies targeting the inhibitory immune checkpoint factors such as programmed death ligand-1 (PD-L1) (nivolumab and pembrolizumab) and cytotoxic T-lymphocyte antigen-4 (CTLA-4) (ipilimumab), oncolytic viruses, peptide vaccine trials, and dendritic cell vaccines to name a few (for a detailed review on immunotherapy see [257]). Given the promising results with immunotherapeutics in other solid cancers, the results from the ongoing combinatorial immunotherapy trials for glioma patients are eagerly awaited.

One of the biggest challenges in the treatment of GBMs is tumor recurrence and resistance to current therapies. Strong evidence is emerging that suggests GBM tumors have hijacked features of normal CNS development, wherein GSCs are the prime suspects and driving force for tumor growth. During development, spatio-temporally regulated signaling pathways govern proliferation, differentiation, and migration of NPCs. The very same signaling pathways are utilized by GSCs to ensure tumor growth and dissemination. While there has been increased interest in investigating the signaling pathways involved in glioma invasion, further studies are needed to determine the role of GSCs and the microenvironment in promoting invasion. Future studies focused on unraveling the complex interplay between the GSCs and the tumor microenvironment will shed light on the signaling pathways and key molecular players that could be targeted for GBM therapy.

## Abbreviations

AMPAR: AMPA receptors
B2R: Bradikynin 2 receptor
bHLH: Basic helix–loop–helix
c-MET: Tyrosine protein kinase Met/hepatocyte growth factor receptor
CaMKII: Ca^2+^/calmodulin-dependent protein kinase II
ClC3: Cl^−^ channel protein 3
CNS: Central nervous system
COL5A1: Collagen 5A1
CPE: Carboxypeptidase E
CSF-1: Colony stimulating factor 1
ECM: Extracellular matrix
EGFR: Epidermal growth factor receptor
EMT: Epithelial-to-mesenchymal transition
FAK: Focal adhesion kinase
FZD: Frizzled receptor
GBM: Glioblastoma
GDNF: Glial-derived neurotrophic factor
GPCRs: G protein-coupled receptors
GSC: Glioma stem cell
HA: Hyaluronic acid
HGF: Hepatocyte growth factor
ICAM-1: Intercellular adhesion molecule-1
IP3R3: Inositol-1,4,5-trisphosphate receptor 3
MAPK: Mitogen-activated protein kinases
MCP-1/CCL2: Monocyte chemoattractant protein 1
miRNA: MicroRNA
MMP: Matrix metalloproteinase
NPCs: Neural stem/progenitor cells
OPC: Oligodendrocyte progenitor cell
PI3K/AKT: Phosphatidylinositol 3-kinase/protein kinase B
OPC: Oligodendrocyte progenitor cell
RHAMM: Receptor for hyaluronate-mediated motility
SDF-1: Stromal cell-derived factor-1
TAM: Tumor-associated macrophage
TβRI/II: TGF-β-receptor type I/II
TCGA: The Cancer Genome Atlas
TF: Transcription factor
TGF-β: Transforming growth factor-β
TNC: Tenascin C
VEGF: Vascular endothelial growth factor
WNT: Wingless/Int1
